# Enhanced Responsivity and Optoelectronic Properties of Self-Powered Solar-Blind Ag_2_O/β-Ga_2_O_3_ Heterojunction-Based Photodetector with Ag:AZO Co-Sputtered Electrode

**DOI:** 10.3390/nano13071287

**Published:** 2023-04-06

**Authors:** Younghwa Yoon, Sangbin Park, Taejun Park, Hyungmin Kim, Kyunghwan Kim, Jeongsoo Hong

**Affiliations:** Department of Electrical Engineering, Gachon University, 1342 Seongnamdaero, Seongnam 13120, Republic of Korea

**Keywords:** β-Ga_2_O_3_, DUV, photodetector, Co-sputtered electrode, Ag:AZO

## Abstract

A Ag:AZO electrode was used as an electrode for a self-powered solar-blind ultraviolet photodetector based on a Ag_2_O/β-Ga_2_O_3_ heterojunction. The Ag:AZO electrode was fabricated by co-sputtering Ag and AZO heterogeneous targets using the structural characteristics of a Facing Targets Sputtering (FTS) system with two-facing targets, and the electrical, crystallographic, structural, and optical properties of the fabricated thin film were evaluated. A photodetector was fabricated and evaluated based on the research results that the surface roughness of the electrode can reduce the light energy loss by reducing the scattering and reflectance of incident light energy and improving the trapping phenomenon between interfaces. The thickness of the electrodes was varied from 20 nm to 50 nm depending on the sputtering time. The optoelectronic properties were measured under 254 nm UV-C light, the on/off ratio of the 20 nm Ag:AZO electrode with the lowest surface roughness was 2.01 × 10^8^, and the responsivity and detectivity were 56 mA/W and 6.99 × 10^11^ Jones, respectively. The Ag_2_O/β-Ga_2_O_3_-based solar-blind photodetector with a newly fabricated top electrode exhibited improved response with self-powered characteristics.

## 1. Introduction

Ultraviolet (UV) radiation plays an important role in various fields such as practical life, the military industry and environment, security applications, the chemical industry, and biology [1,2]. Of the total solar ultraviolet photons, deep-UV (DUV) light with a wavelength of less than 280 nm is absorbed by the ozone layer, which is the main region that absorbs the corresponding photons before they reach the surface of the earth. It is called a solar-blind UV light (200–280 nm) and has extremely low background noise and high responsibility [3]. Therefore, solar-blind UV photodetectors using these unique properties were developed and used extensively in medical imaging, chemical and biological analysis, flame sensor, and military-related industries, such as secure fields and missile detection [4,5,6,7,8].

Attempts were made to find suitable materials for the fabrication of a solar-blind photodetector, and a wide bandgap material of 4.42 eV or more to detect and measure UV light with a wavelength of <280 nm was required as a top priority. Studies have shown that Ga_2_O_3_ has several advantages as a material for the fabrication of solar-blind UV light photodetectors. 

Ga_2_O_3_ has an ultra-wide band gap of 4.7–5.2 eV in bare state, excellent thermal and chemical stability, and high selectivity to UV light [9,10,11]. Owing to its great potential, many studies of the Ga_2_O_3-_based solar-blind UV photodetector have been conducted recently [12,13,14].

Photovoltaic Ga_2_O_3_ solar-blind photodetectors are being developed in the form of Schottky, PN, and PIN photodetectors [15]. However, solar-blind photodetectors are applied in extreme environments that require continuous and long-term use without a separate power supply and stability against corrosion and temperature. For this reason, a p-n junction photodetector is advantageous for self-powered photodetectors that use the built-in electric field of photovoltaic devices by transferring the photo-excited electron-hole pairs to generate an electrical signal with a fast response speed [16,17,18,19,20,21].

Recent research has shown that various materials and structures can be used to enhance photodetection performance. For instance, Ga_2_O_3_/zero-dimensional graphene quantum dot (GQD) heterojunction, Ga_2_O_3_/MXene nanowire networks, MSM structured ZnO/Au/ Ga_2_O_3_, and multi-layer thin film have all been applied to improve photodetection performance [22,23,24,25,26].

In a previous study, our group fabricated a Ag_2_O/β-Ga_2_O_3_ p-n junction-based photodetector to increase photo-excited electron-hole pair transfer efficiency, which led to an improvement in key parameters such as responsivity [27]. Our study also observed differences in optical performance based on the thickness and roughness of the electrode. From these results, we identified the importance of the upper electrode used in the photodetector and the need for further research in this area.

Therefore, we designed a new electrode fabrication with low roughness from representative thin film materials, such as silver (Ag), aluminum-doped zinc oxide (AZO), copper (Cu), gold (Au), and graphene [28,29,30]. We fabricated a novel electrode material that combines the properties of Ag and AZO, which was expected to show a uniform thin film and low surface roughness, based on research on AZO/Ag/AZO multilayer thin film [31]. Although the effect of AZO on the formation of low roughness has not yet been studied, it is expected that the use of AZO with Ag will result in a low roughness surface of the electrode, just as AZO has been shown to assist in the formation of ultra-thin Ag films in studies on AZO/Ag/AZO thin films. Therefore, a thin film was deposited by the co-sputtering of Ag and AZO to create a Ag:AZO thin film, where the composition ratio of Ag and AZO was adjusted.

A co-sputtering technique using the Facing Targets Sputtering (FTS) system was applied to the deposition of a Ag:AZO thin film, which enabled the production of material compositions that are technically impossible to produce as a single target [32,33,34]. Moreover, a low-resistance, high-conductivity, and transparent Ag:AZO thin film was fabricated by controlling the co-sputtering conditions. Ag:AZO, Ag, and AZO electrodes were deposited on the Ag_2_O layer of the Ag_2_O/β-Ga_2_O_3_ heterojunction photodetector, and the photo responses of each photodetector were compared and evaluated. The thickness of each electrode was varied between 20 and 50 based on the sputtering time. The optoelectronic properties were measured under a 254 nm UVC light, and the Ag:AZO electrode exhibited a photoresponsivity of 56 mA/W without any additional voltage. The Ag_2_O/β-Ga_2_O_3-_based solar-blind photodetector using a Ag:AZO upper electrode exhibited enhanced responsivity with the self-powered property.

## 2. Experiment

### 2.1. Materials

The fabrication of the p-n photodetector was accomplished using an n-type Sn-doped β-Ga_2_O_3_ wafer (N_d_–N_a_ = 6.3 × 10^18^ cm^–3^ with a thickness of 641 μm, Novel Crystal Technology, Inc., Saitama, Japan). For the epitaxial layer, Si-doped β-Ga_2_O_3_ (N_d_–N_a_ = 2.8 × 10^16^ cm^–3^ with a thickness of 8.6 μm, Novel Crystal Technology, Inc., Japan) was grown using halide vapor phase epitaxy. For the ohmic contact of the device, Ti/Au electrodes (10/40 nm) were deposited by electron-beam evaporation on the backside of the Sn-doped β-Ga_2_O_3_ wafer. Targets of 4-inch Ag and ZnO:Al_2_O_3_ (98:2 wt%) (RND Korea, Seoul, Republic of Korea) were purchased for the deposition of p-type Ag_2_O and upper electrodes. The soda-lime glass substrate (size of 75 × 25 × 1 mm^3^, Marienfeld, Lauda-Königshofen, Germany) was used in the experiment for measuring the characteristics of electrodes.

### 2.2. Fabrication and Characterization of the Ag:AZO Thin Film

**Scheme 1:**
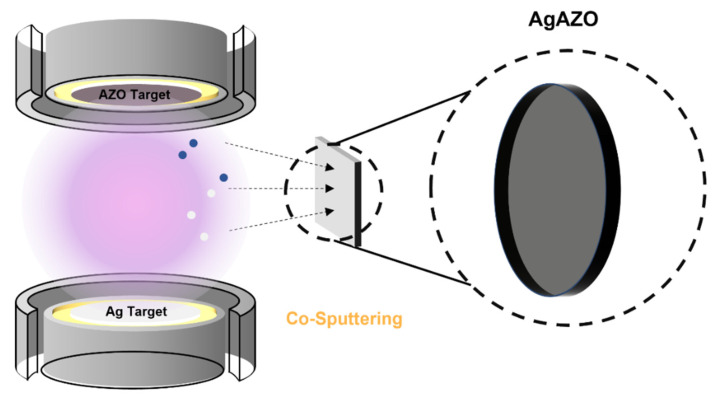
The deposition of Ag:AZO thin film by co-sputtering method.

As presented in Scheme 1, the co-sputtering method was applied with an FTS system to prepare Ag:AZO samples on soda-lime glass. The thin film was deposited under the sputtering conditions presented in Table 1, and the thickness of the thin film was measured by a KLA-Tencor α-Step D-500 Stylus Profiler and then deposited according to the calculated deposition rate shown in Table 2. The properties of Ag:AZO as an electrode material were evaluated, and the Ag and AZO samples were also evaluated after deposition. The X-ray diffraction (XRD) patterns were measured on a diffractometer (PANalytical; X’Pert Pro MPD, Almelo, The Netherlands). The surface morphologies of the films were observed using scanning electron microscopy (SEM; S-4700, Hitachi, Tokyo, Japan) and atomic force microscopy (AFM; Park NX10, Park Systems, Suwon, Republic of Korea). The optical properties were evaluated by a UV-visible spectrophotometer in the range of 190–800 nm (Lambda 750 UV-vis-near-infrared, PerkinElmer, Waltham, MA, USA).

### 2.3. Fabrication and Evaluation of Ag_2_O/β-Ga_2_O_3_-Based Photodetector with Different Electrodes

**Scheme 2:**
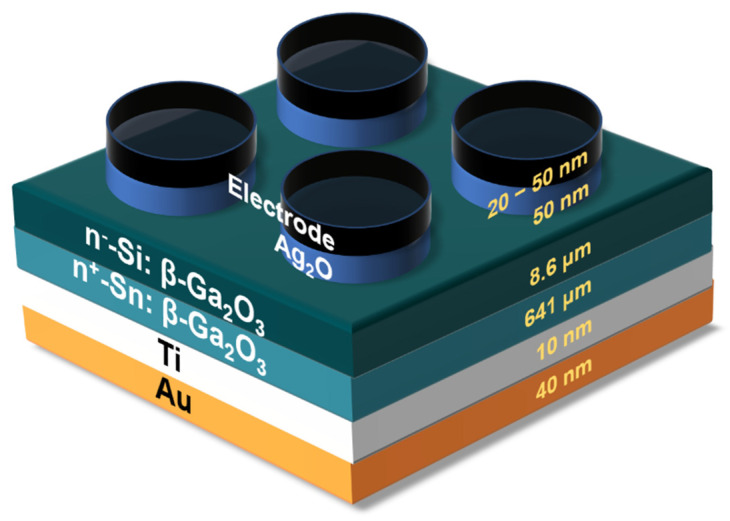
Schematic illustration of the p-n junction photodetector.

Scheme 2 shows the as-fabricated p-n junction photodetector. The 4 inch Ag targets were used to deposit the p-type Ag_2_O layer on the top of the β-Ga_2_O_3_ epitaxial layer [35]. As shown in Table 3, the sputtering conditions were controlled during the deposition with FTS in an oxygen-saturated atmosphere. Each electrode was deposited in a circular shape on the Ag_2_O layer by using a shadow mask with a radius of 300 μm. To enhance crystallization and reduce the interfacial defect of Ag_2_O, the as-fabricated samples were post-annealed at 300 °C for 1 min at 100 mTorr in an Ar atmosphere using rapid thermal annealing. Electrical characteristics such as current-voltage (I–V) ratio and photosensitivity were measured and analyzed with a semiconductor analyzer (4200A-SCS Parameter Analyzer, Keithley, Cleveland, OH, USA) in response to various light intensities using a UV-C lamp (TN-4LC, wavelength: 254 nm, Korea Ace Scientific, Seoul, Republic of Korea).

## 3. Results and Discussion

### 3.1. Evaluation of As-Deposited Electrodes

In our previous study, Ag was used as the upper electrode material for the Ag_2_O/β-Ga_2_O_3_-based p-n junction photodetector. This study confirmed that the surface condition of the electrode film has a significant influence on the performance of the photodetector. Therefore, as a further study, we tried to fabricate an electrode with an improved surface and apply it to the device. Therefore, a thin film was made by co-sputtering Ag and AZO, and the structural, optical, and electrical properties of the as-deposited thin film on the glass substrate were evaluated.

**Figure 1:**
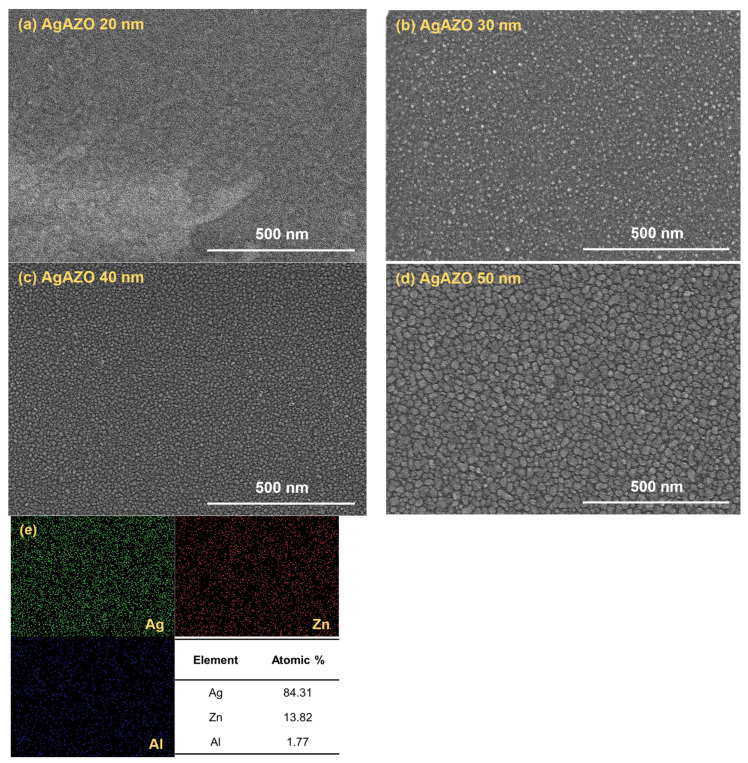
Thickness-dependent SEM images of Ag:AZO thin films. (**a**) 20 nm; (**b**) 30 nm; (**c**) 40 nm; (**d**) 50 nm; (**e**) EDX image of 20 nm Ag:AZO thin film.

The properties of the electrodes were evaluated prior to deposition on the Ag_2_O/β-Ga_2_O_3_ photodetector. Surface morphology in thin films analyzed by SEM are described in Figure 1a–e and Figures S1 and S2. The SEM images shown in Figure 1a–d are 20–50 nm thick Ag:AZO films deposited by co-sputtering with Ag and AZO. The films exhibited smooth and homogeneous surfaces, and grain boundaries increased with increasing thickness. The uniformity of the surface improves photosensitivity when used as a layer of a photodiode device by reducing the scattering and reflectivity of the irradiated light. Figure 1e is an energy-dispersive X-ray spectrometer (EDX) image of a 20 nm sample deposited at 50 W, showing the distribution and atomic percentages of Ag, Al, and ZnO present in the thin film. Since AZO is an oxide, it has a lower deposition rate than Ag, and it is inferred that more Ag is contained in the component table of the as-fabricated sample. Figures S1 and S2 show the surface SEM images according to the thickness of Ag and AZO thin films, respectively. It was observed to have a homogeneous surface like that of the Ag:AZO thin film.

**Figure 2:**
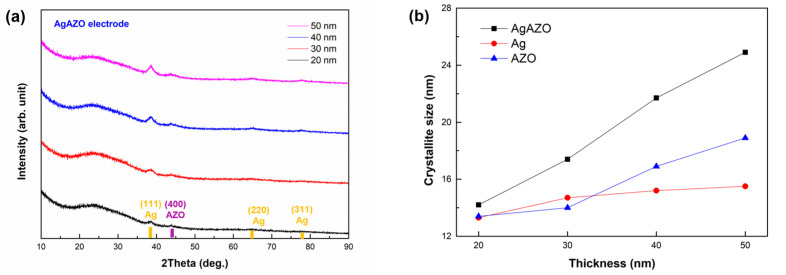
Structural characteristics of Ag:AZO thin film depending on thickness. (**a**) XRD measurement results. (**b**) Crystallite size according to the thickness of the electrodes.

The crystallographic characteristics of the as-fabricated Ag:AZO film for each thickness were evaluated by analyzing the XRD patterns in Figure 2a. The Ag:AZO samples had polycrystalline structures, and both Ag and AZO peaks were observed. Ag peaks at 2θ = 38.12, 64.4, and 77.5° corresponded to the (111), (220), and (311) planes, respectively, and the AZO peak at 2θ = 44.8° corresponded to the (400) plane (Ag; ICDD card 01-087-0720, AZO; ICDD card 01-071-0968). There was no significant change based on thickness; however, compared with the peak intensity of the AZO electrode in Figure S3, the (400) peak intensity was lower because the AZO content in the Ag:AZO sample was 5.6 times lower than that of Ag, as we inferred from the EDX results.

The crystallite size was calculated using the Scherrer equation based on the (111) plane, which is the preferential growth plane of the XRD pattern of Ag:AZO, and it was inferred that the crystallite size depends on the thickness. The Scherrer equation is as follows [36,37]:

(1) $$\tau =\frac{K\lambda }{\beta cos\theta }$$

In Equation (1), 𝜏 is the crystallite size, *θ* is the Bragg angle, *K* is the Scherrer constant, and 𝛽 is the full width at half maximum (FWHM) inversely proportional to the crystallite size 𝜏. Therefore, the larger the FWHM, that is, the wider the XRD pattern, the smaller the crystallite size. The crystallite size according to the thickness of the thin films is shown in Figure 2b. As the thickness increased, so did the crystallite size, and it was inferred that Ag:AZO, which had the smallest FWHM, had the largest crystallite size. Crystallite size increases as the density of a material increases; hence, a flat surface would be made of dense crystallites.

**Figure 3:**
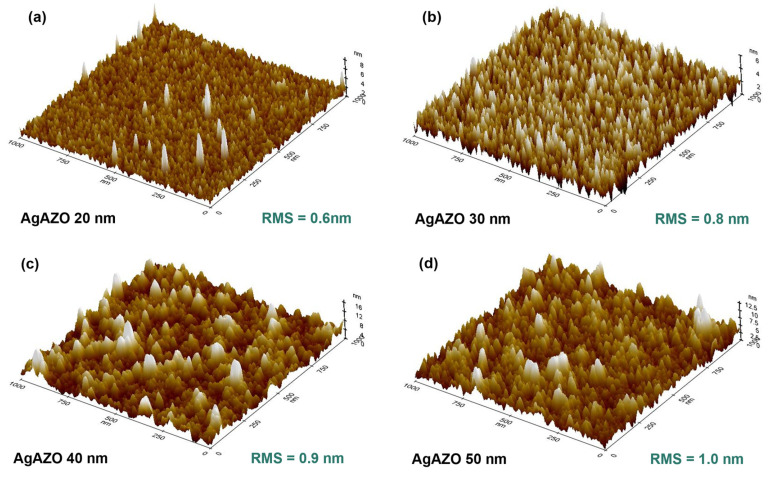
AFM images of the Ag:AZO thin film as a function of thickness. (**a**) 20; (**b**) 30; (**c**) 40; (**d**) 50 nm.

The low roughness of the thin film is an important factor for electrodes used in photodetectors, as it reduces loss such as scattering and reflection of incident light energy [38]. Therefore, the roughness of each thickness of the deposited samples was obtained using AFM in the range of 1 × 1 μm^2^ images and is shown in Figure 3, Figures S4 and S5. The RMS values representing the surface roughness of the Ag:AZO thin film ranged from 0.6 to 1.0 nm (20–50 nm Ag:AZO thin film). However, the range of RMS values of the Ag and AZO thin films were 1.0 to 1.3 nm and 0.9 to 1.2 nm, respectively, indicating that the Ag:AZO thin film had the lowest roughness compared with the same thickness. The roughness of the Ag:AZO 20 nm thin film was reduced by 25% compared with the previously used Ag 20 nm thin film. The roughness of the thin film is a similar feature to the crystallite size calculated by the Scherrer equation based on the XRD pattern, and by this, it can be inferred that a smoother surface is formed in the thin film with a large crystallite size. In addition, because the roughness of the thin film has a great effect on the performance of the photodetector, it is expected that a photodetector with high responsivity can be manufactured using the Ag:AZO electrode.

**Figure 4:**
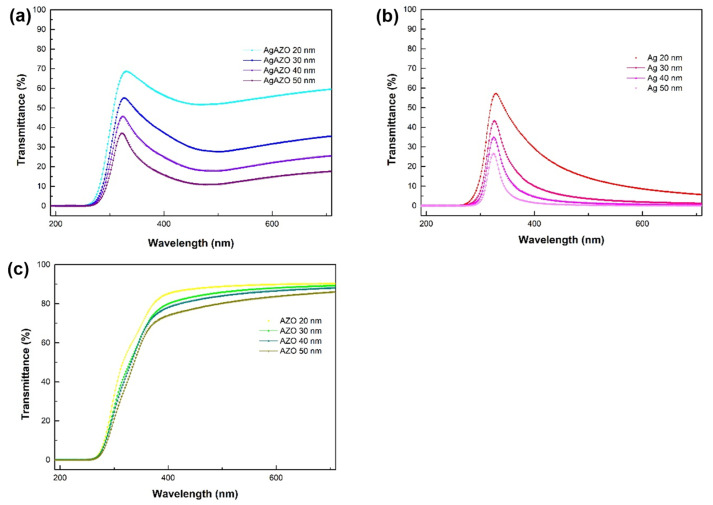
Optical characteristics of as-deposited electrodes by thickness. (**a**) Transmittance of Ag:AZO thin film (**b**) Transmittance of Ag thin film (**c**) Transmittance of AZO thin film.

Figure 4 shows the transmittance of the fabricated thin films in the wavelength range of 190 to 800 nm using a spectrophotometer. AZO, a transparent electrode, exhibited a high transmittance in visible light and a transmittance of 89% at a thickness of 20 nm. However, Ag exhibited a peak transmittance of 57% in the 320 nm wavelength band, and a low transmittance in the other wavelength bands except for the corresponding region. The Ag:AZO thin film fabricated via the co-sputtering of Ag and AZO exhibited optical properties of both Ag and AZO, and had a peak at 320 nm. It also exhibited a transmittance of 55 to 70% at a thickness of 20 nm.

The Hall meter was used to evaluate the electrical properties of the fabricated thin film to be used as an electrode. As shown in Table 4, the sheet resistance, carrier concentration, and mobility were inferred, and after application to the device, changes in electrical properties were also observed after heat treatment of the electrode for post-heat treatment at 300 °C, to improve the crystallinity of Ag O [39,40,41].

The sheet resistance of various thicknesses of deposited thin films decreased as the thickness increased, and in the case of the Ag:AZO thin film, the sheet resistance was at least six times lower than that of the AZO thin film, which is an existing electrode material, at all thicknesses. In addition, after annealing at 300 °C, the sheet resistance of the 20 nm Ag:AZO thin film was reduced by half from 72.8 to 38.1 Ω/Sq. Regarding carrier concentration with respect to temperature change, all three electrodes exhibited an increasing change, but there was a difference in the increase rate depending on the electrode. This increase in carrier concentration is because defects generated during thin film deposition using sputtering are reduced via heat treatment, thereby preventing loss due to trapping during the movement of carriers [42]. However, owing to the increased electron concentration, the hole mobility decreased.

As shown in Table 5, it was confirmed how the work function of Ag:AZO and co-sputtered Ag:AZO changed by measuring the work function of the deposited electrode. The work function of Ag:AZO is 4.66 eV, which is higher than Ag: 4.26 eV and AZO: 4.1 eV.

### 3.2. Characteristics of As-Deposited P-Type Ag_2_O Thin Film

**Figure 5:**
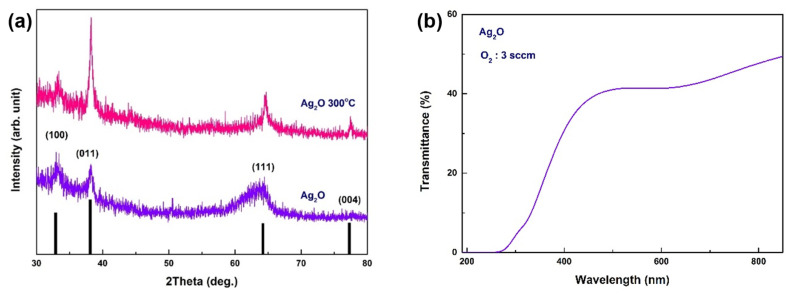
Characteristics of p-type Ag_2_O thin film. (**a**) XRD pattern of Ag_2_O thin film. (**b**) Transmittance of Ag_2_O thin film.

P-type Ag_2_O thin films were fabricated using reactive sputtering in an oxygen atmosphere. To create an oxygen atmosphere in the chamber, gas was introduced at an Ar to O_2_ ratio of 10:3 sccm, and sputtering was performed at an applied power of 50 W. Figure 5a is the XRD result of the deposited Ag_2_O thin film. Peaks appearing in the XRD pattern are as follows. The Ag_2_O peaks at 2θ = 33.66, 36.34, 63.36, and 66.74° corresponded to the (100), (011), (111), and (004) planes, respectively. (Ag_2_O; ICDD card. 01-072-2108). After the heat treatment, the intensity of the (011) peak increased, indicating that the crystallinity of the preferentially grown surface of the Ag_2_O thin film improved. In addition, it was inferred that the (004) plane, which was a fine peak around 66.74° after heat treatment, greatly increased [43]. The Ag_2_O particle size calculated from the XRD results was 13.3 nm. The electrical properties of Ag_2_O measured by a Hall meter were carrier concentration, mobility, and resistivity, measuring 6.35 × 10^18^ cm^−3^, 71.4 cm^2^/Vs, and 1.38 × 10^−2^ Ω·cm, respectively. In addition, the fabricated sample was measured by UV-vis and exhibited a transmittance of 40%, as shown in Figure 5b.

### 3.3. Characteristic Evaluation of Fabricated Device

**Scheme 3:**
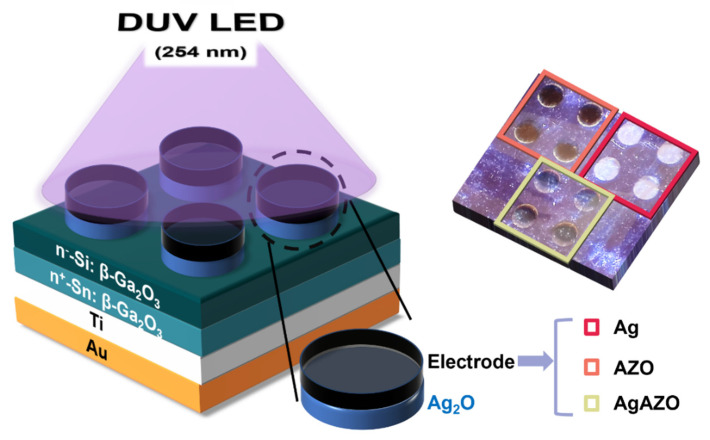
Schematic diagram of a photodetector with three different electrodes applied.

Scheme 3 is a photodetector fabricated by sequentially depositing the p-type Ag_2_O and electrodes evaluated above on a β-Ga_2_O_3_ substrate. As shown in the figure, all three electrodes with the same thickness were deposited on one device to evaluate the semiconductor characteristics and photoreaction.

**Figure 6:**
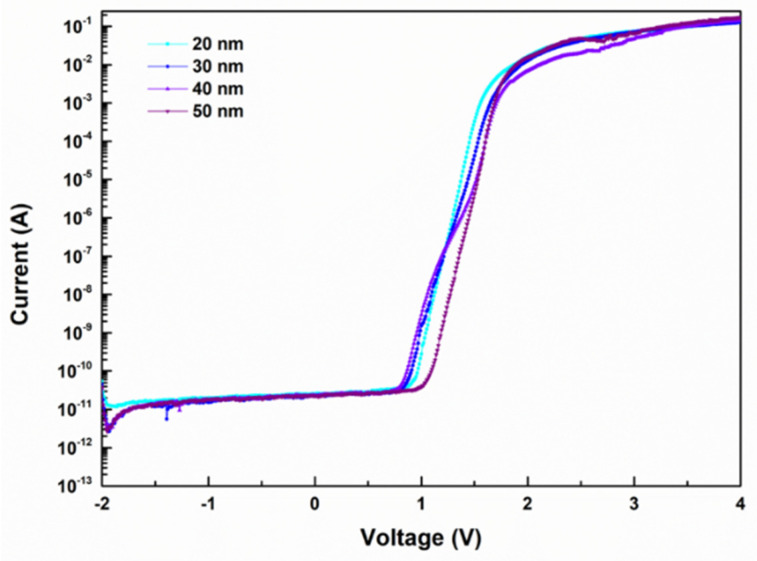
I-V curve of photodetector using Ag:AZO electrode.

Figure 6 is the I–V curve for each thickness of a Ag_2_O/β-Ga_2_O_3_ photodetector based on the p-n junction deposited with a Ag:AZO electrode. Measurements were conducted in a dark room to eliminate the influence of light, and voltages from −2 to 4 V were applied to evaluate current changes according to voltage. The rectification characteristics of the p-n junction were observed at all thicknesses, and the off- and on-currents of the Ag:AZO 20 nm device were 2.56 × 10^−11^A at 0 V and 5.16 × 10^−3^ A at 1.73 V, respectively. The on/off ratio was calculated as 2.01 × 10^8^.

**Figure 7:**
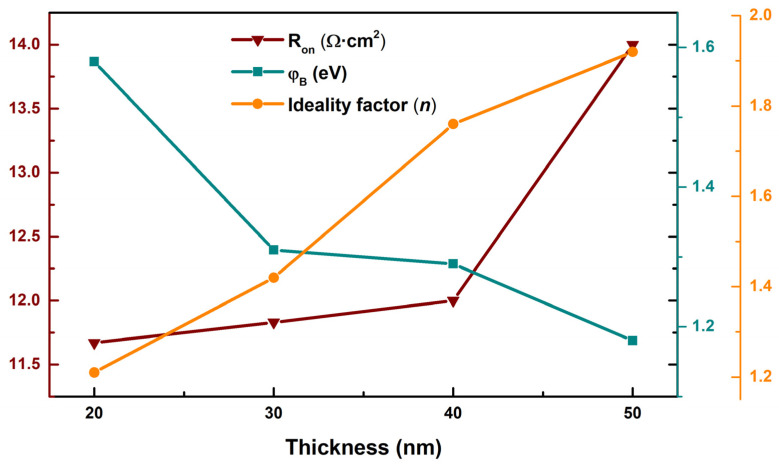
Electrical parameters of the photodetector as a function of Ag:AZO thickness.

Figure 7 shows the parameters calculated from the I–V curve of the photodetector shown in Figure 5 based on the thermionic emission model [44]. The parameters were on-resistance (R_on_), barrier potential (*φ*_B_), and ideality factor (*n*) based on thickness, and the thermionic emission model is as follows [45].

(2) $$J=J_{s}\exp\left\{\frac{q\left(V-IR_{s}\right)}{nkT}\right\}$$

(3) $$J_{s}=AA^{*}T^{2}\exp\left(\frac{-q\varphi_{B}}{kT}\right)$$

In Equation (2), *J_s_* is the saturation current density, *q* is the charge, *V* is the voltage across the diode, *R_s_* is the series resistance, and *n* is the ideality coefficient representing the deviation between the ideal diode and the actual non-uniform barrier and tunneling diode. *k* is the Boltzmann constant and *T* is the Kelvin temperature. In Equation (3), A is the contact area, A* is Richardson’s constant, and β-Ga_2_O_3_ has a value of 41 A/(cm^2^*K^2^).

As the electrode thickness increased, the on-resistance increased from 11.67 to 14.0 Ω·cm^2^, the ideality coefficient increased from 1.42 to 2.42, and the barrier potential decreased from 1.58 eV to 1.29 eV. The change in the ideality factor is related to the interface state between the electrode and the p-type layer. As the thickness of the electrode increased, the stress and roughness of the material increased, causing defects at the interface between the p-type and the electrode, which hindered the capture or movement of carriers, leading to an increase in resistance [46]. However, it was inferred that the barrier height increased as the thickness decreased because the roughness and interface defects decreased as the thickness of the electrode decreased. Defects present in the interfacial layer degrade the movement of carriers, so carriers move smoothly in samples with few defects, forming a wide depletion layer; hence, the barrier height increased [47]. A photodetector with a high ideality coefficient is affected by charge trapping and carrier recombination, so it was judged that the device with the 20 nm Ag:AZO electrode had the best electrical characteristics [48]. In addition, one of the causes of a high ideality coefficient is the hump phenomenon present in the I–V curve, which affects the current flow because of the high trap density due to the trapping of charged species in Ag and AZO combined crystals with different resistivity and conductivity. It was inferred that the hump phenomenon had occurred.

**Figure 8:**
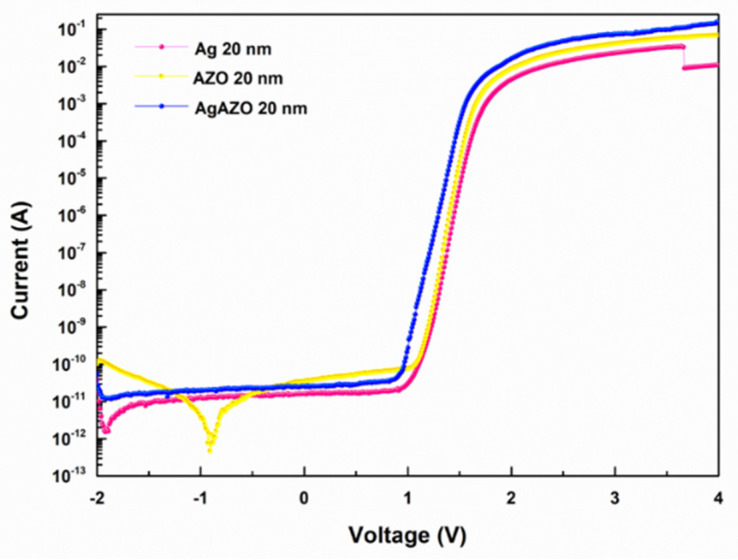
Photodetector I–V curves using 20 nm-thick electrodes.

Figure 8 shows I–V curves of three electrodes with thicknesses of 20 nm. Rectification characteristics of the p-n junction were observed in all devices using each electrode, and the off- and on-currents of the Ag device were 1.62 × 10^−11^ A at 0 V and 6.23 × 10^−4^ A at 1.71 V, respectively, with an on/off ratio of 3.85 × 10^7^. In addition, the off- and on-currents of the AZO device were found to be 2.58 × 10^−11^ A at 0 V and 7.03 × 10^−4^ A at 1.71 V, respectively, resulting in an on/off ratio of 2.72 × 10^7^. Therefore, the Ag:AZO electrode was found to have the largest on/off ratio. Table 6 displays the photodetector’s electrical parameters for each electrode. AZO, as an oxide, has a higher resistance than other electrodes, resulting in a relatively high *R_on_* value compared with the other electrodes. However, it can be inferred that there are few interface defects with the p-type layer, as the ideality coefficient is closest to one and the potential barrier is high.

**Figure 9:**
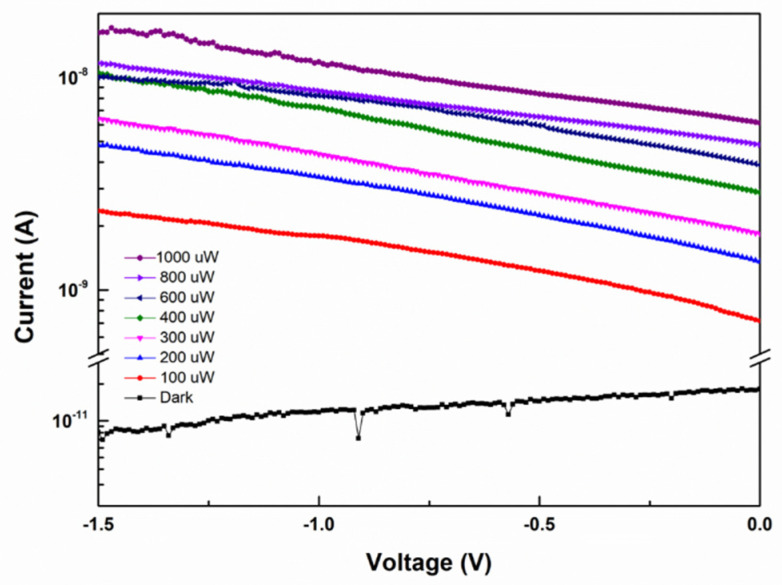
I–V curve of Ag:AZO 20 nm device at different UV light intensities.

Figure 9 depicts the I–V curve as a function of the intensity of UV irradiation on the Ag:AZO 20 nm device. Reverse bias is generated by applying irradiating light with a forward bias to the device, and reverse current is generated because of the generated electron-hole pair. Therefore, as shown in the figure, it was inferred that the stronger the light intensity, the greater the reverse current.

**Figure 10:**
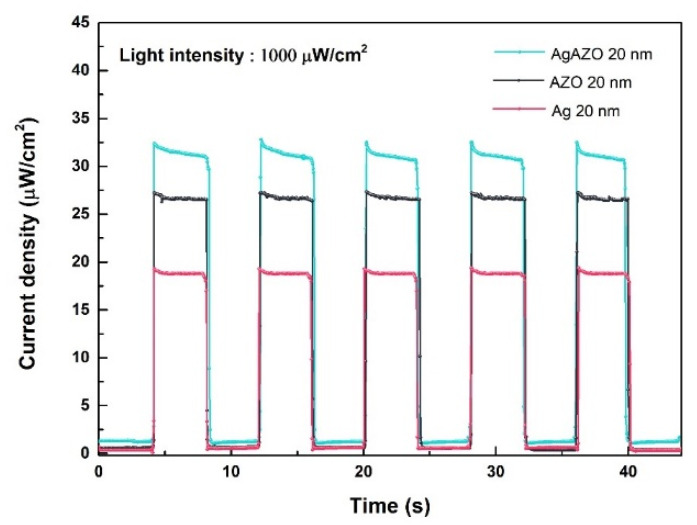
Photocurrent density of each device for 1000 μW/cm^2^ UV light.

Figure 10 shows the photocurrent densities of the photodetectors to which each electrode was applied over time, when irradiated with UV radiation with an intensity and wavelength of 1000 μW/cm^2^ and 254 nm, respectively, in zero bias. Ag:AZO exhibited the highest photocurrent density of 31 μA/cm^2^, compared with AZO and Ag, which had photocurrent densities of 27 μA/cm^2^ and 18.6 μA/cm^2^. There are two reasons for this phenomenon. First, because of the low surface roughness, loss due to scattering and the reflection of light energy incident on the surface of the thin film is relatively small. Second, because of the low roughness of the electrode, sufficient bonding between the interface layers is achieved, resulting in lower contact resistance. Therefore, it is expected that the largest photocurrent appears in the photodetector using the 20 nm Ag:AZO electrode with the lowest roughness.

**Figure 11:**
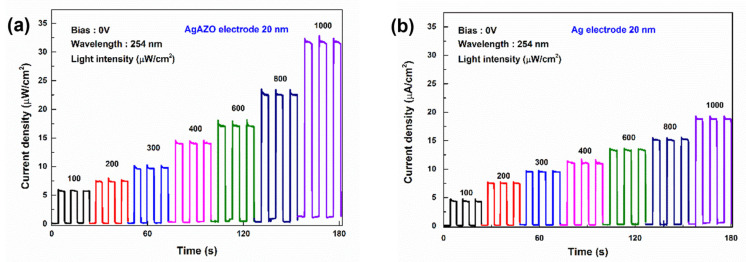

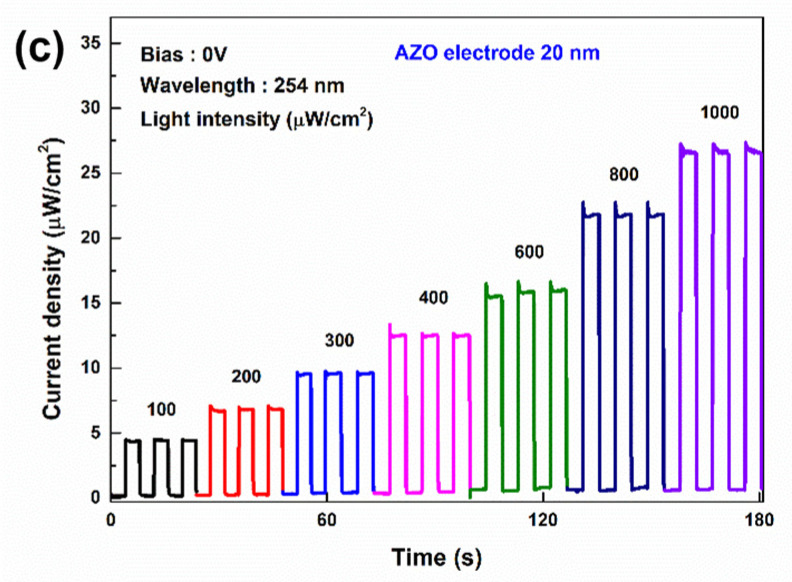
Photocurrent density change of device according to light intensity. (**a**) Ag:AZO 20 nm; (**b**) Ag 20 nm; (**c**) AZO 20 nm.

**Figure 12:**
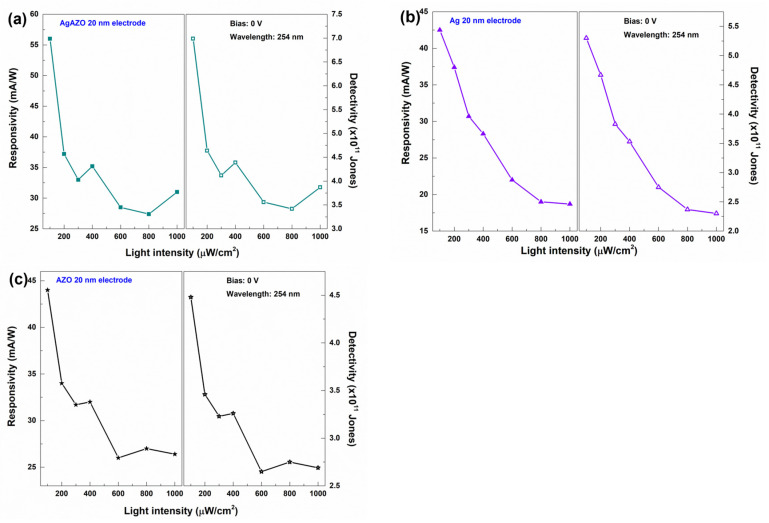
Device responsivity and detectivity change according to light intensity. (**a**) AgAZO 20 nm; (**b**) Ag 20 nm; (**c**) AZO 20 nm.

In Figure 11, the photocurrent density obtained by irradiating each element with UV from 100 to 1000 μW/cm^2^ in zero bias is shown. As the light intensity increased, more electron-hole pairs were generated, and the photocurrent increased. The light intensity-photocurrent density of the devices exhibited a linear increase. Responsivity, which represents photosensitivity and is an important index for evaluating the performance of photodetectors, and detectivity, which is a performance index for the smallest detectable signal, were evaluated from the photocurrent density value based on the light intensity. Figure 12 shows the responsivity and detectivity as a function of the light intensity of each device. The equations for calculating responsivity and detectivity are as follows [49,50,51,52].

(4) $$R=\left(\frac{J_{photo}-J_{dark}}{P}\right)$$

(5) $$D=\frac{R}{{\left(2eJ\right)}^{1/2}}$$

In Equation (4), *J_photo_* is the photocurrent density, *J_dark_* is the dark-current density, and *P* is the irradiated-light intensity. In Equation (5), *e* is the absolute value of charge, and *J* is the dark-current density. The responsivity and detectivity of each device calculated with the above formulas are shown in Figure 12. Responsivity and detectivity decreased as the intensity of light increased. At higher intensities, more electron-hole pairs were generated, which induced self-heating due to collisions and vibrations between electrons, increasing charge carriers, as well as increasing recombination rates. Therefore, the highest value was measured at 100 μW/cm^2^, where the effect of self-heating was the least. The maximum values of responsivity and detectivity of each photodetector with 20 nm electrode applied were Ag:AZO: 56 mA/W, 6.99 × 10^11^ Jones; Ag: 42.5 mA/W, 5.3 × 10^11^ Jones; and AZO: 44.4 mA/W, 4.8 × 10^11^ Jones. In order to check the response spectra of the photodetector, the same measurement was performed under 365 nm light, but no photo-response was observed

**Figure 13:**
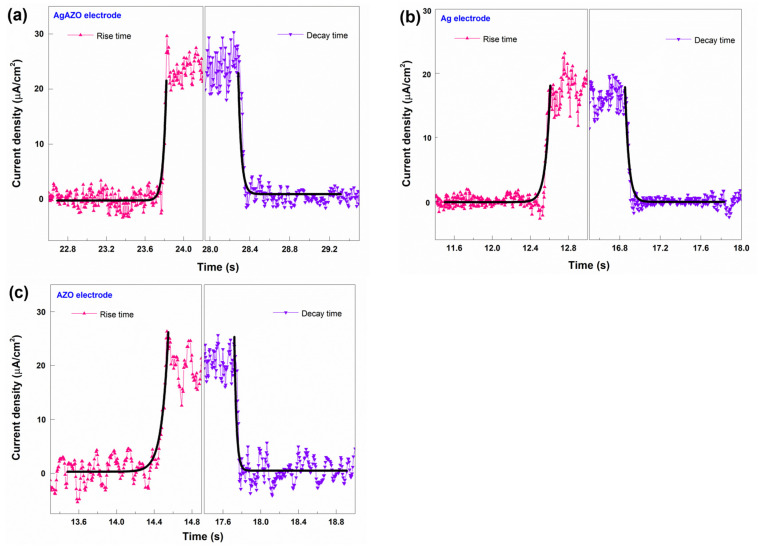
Rise time and decay time of time-dependent photocurrents of each photodetector irradiated with 1000 μW/cm^2^ UV light. (**a**) Ag:AZO 20 nm; (**b**) Ag 20 nm; (**c**) AZO 20 nm.

Rise and decay times, which evaluate the response speed of the device, are shown in Figure 13. Response speed, one of the important factors in evaluating photodetectors, is determined by the size of the internal potential caused by the difference in band gap between p-type Ag_2_O and n-type β-Ga_2_O_3_ and measured using photocurrent characteristics over time [50,53,54,55,56,57,58,59]. When a UV radiation of 1000 μW/cm^2^ was used in zero bias, the rise and fall times of the Ag:AZO, Ag, and AZO devices were 31.4 and 22 ms, 26 and 43 ms, and 63 and 63.8 ms, respectively. Based on the characteristics of the fabricated photodetector, the photodetector using the 20 nm-thick Ag:AZO electrode with the lowest surface roughness exhibited the highest photo response and responsivity. Furthermore, based on the current flowing characteristics, it was inferred that the p-n junction structure was driven without an external power supply owing to the built-in potential [60,61]. As shown in Figure S6, additional measurement with a light of 1000 μW/cm^2^ was conducted to confirm the sustainability of the Ag:AZO photodetector after 6 months of fabrication.

## 4. Conclusions

The structural, optical, and crystallographic properties of the Ag:AZO electrode fabricated via co-sputtering using FTS were evaluated, and it was inferred that it had the lowest roughness at 20 nm. It was determined that the thin film with low roughness would improve photodetector performance by reducing light energy loss and the interfacial trap phenomenon. A Ag_2_O/β-Ga_2_O_3_ photodetector was fabricated. The on/off ratio of the device was 2.01 × 10^8^, and its responsivity and detectivity were 56 mA/W and 6.99 × 10^11^ Jones, respectively. The result was better than that of the previously used Ag and AZO electrodes, and similar features to the surface roughness value of each electrode were obtained. In addition, the rise and fall times of the Ag:AZO device under UV irradiation of 1000 μW/cm^2^ in zero bias were 31.4 and 22 ms, respectively. Consequently, the performance of the photodetector was improved by using a thin film with low roughness to reduce the defect, which decreased the photocurrent owing to a large amount of light scattering and reflection on the surface of the film.

## Figures and Tables

**Table 1:** Sputtering conditions of each electrode.

Parameters	Sputtering Conditions		
	Ag	AZO	Ag:AZO
Targets	Ag (99.99%)	AZO (98:2 wt%)	Ag, AZO
Base pressure (Torr)	3 × 10^−5^	3 × 10^−5^	3 × 10^−5^
Working pressure (mTorr)	2	1	2
Gas flow (sccm)	Ar: 10	Ar: 10	Ar: 10
Input power (W)	50 (DC)	150 (DC)	50 (DC)
Thickness (nm)	20, 30, 40, 50		

**Table 2:** Deposition rate of each electrode by thickness.

Targets	20 nm	30 nm	40 nm	50 nm
Ag	2 min 8 s	3 min 12 s	4 min 16 s	5 min 20 s
AZO	1 min 30 s	2 min 15 s	3 min	3 min 45 s
Ag:AZO	1 min 49 s	2 min 43 s	3 min 38 s	4 min 32 s

**Table 3:** Sputtering conditions of p-type Ag_2_O layer.

Parameters	Sputtering Conditions
Targets	Ag (99.99%)
Base pressure (Torr)	3 × 10^−5^
Working pressure (mTorr)	2
Gas flow (sccm)	Ar: 10, O_2_: 3
Input power (W)	50 (DC)
Thickness (nm)	50

**Table 4:** Electrical properties of as-deposited electrodes.

Ag:AZO	R. T	300 °C	R. T	300 °C	R. T	300 °C
Thickness	Sheet Res. (Ω/Sq.)		Mobility (cm^2^/Vs)		Carrier Concentration (cm^−3^)	
20 nm	38.4	20.4	372	5.09	1.61 × 10^20^	1.61 × 10^23^
30 nm	44.2	26	152.7	3.3	3.08 × 10^20^	2.43 × 10^23^
40 nm	72.8	31.7	153.4	5.14	3.21 × 10^20^	1.49 × 10^23^
50 nm	120.6	142.3	496.9	1.97	2.08 × 10^19^	4.46 × 10^22^
Ag	R. T	300 °C	R. T	300 °C	R. T	300 °C
Thickness	Sheet Res. (Ω/Sq.)		Mobility (cm^2^/Vs)		Carrier Concentration (cm^−3^)	
20 nm	0.952	0.966	4303.3	11.19	7.62 × 10^20^	2.89 × 10^23^
30 nm	0.572	0.515	5462.5	8.84	6.66 × 10^20^	4.57 × 10^23^
40 nm	0.394	0.3	6016.8	4.32	6.58 × 10^20^	1.21 × 10^24^
50 nm	0.289	0.236	6315.7	9.06	6.84 × 10^20^	5.84 × 10^23^
AZO	R. T	300 °C	R. T	300 °C	R. T	300 °C
Thickness	Sheet Res. (Ω/Sq.)		Mobility (cm^2^/Vs)		Carrier Concentration (cm^−3^)	
20 nm	459.4	431.3	69.47	2.59	9.78 × 10^19^	2.79 × 10^21^
30 nm	483.4	383.9	749.1	8.69	5.75 × 10^18^	6.24 × 10^20^
40 nm	441.4	356.8	1405.3	3.63	2.52 × 10^18^	1.21 × 10^21^
50 nm	412.9	370.8	2614.9	3.02	1.16 × 10^18^	1.11 × 10^21^

**Table 5:** Work function of electrodes measured by ultraviolet photoelectron spectroscopy.

Electrode	Work Function
Ag	4.26 eV
AZO	4.1 eV
Ag:AZO	4.66 eV

**Table 6:** Electrical parameters of the photodetector for each electrode.

	Ag:AZO	Ag	AZO
R_on_ (Ω·cm^2^)	11.67	43.33	2170.21
φ_B_ (eV)	1.58	1.68	1.93
*n*	1.21	1.29	1.12

## Data Availability

The data is available on reasonable request from the corresponding author. The data is included in the main text and/or the Supplementary Materials.

## Supplementary Materials

The following supporting information can be downloaded at: https://www.mdpi.com/article/10.3390/nano13071287/s1, Figure S1: Thickness-dependent SEM images of Ag thin films; Figure S2: Thickness-dependent SEM images of AZO thin films; Figure S3: Structural characteristics of Ag:AZO thin film depending on thickness; Figure S4: AFM images of the Ag thin film as a function of thickness; Figure S5: AFM images of the AZO thin film as a function of thickness; Figure S6: Sustainability of Ag:AZO photodetector after 6 months.
