# Supplementary material for: Enhanced Responsivity and Optoelectronic Properties of Self-Powered Solar-Blind Ag2O/β-Ga2O3 Heterojunction-Based Photodetector with Ag:AZO Co-Sputtered Electrode
Source: Nanomaterials (Basel). 2023 Apr 6;13(7):1287. doi: 10.3390/nano13071287 (PMC10096629; doi:10.3390/nano13071287)
Supplement: Supplementary file 1 [file nanomaterials-13-01287-s001.zip › nanomaterials-2265919-supplementary.pdf]

Supplementary Materials

Enhanced Responsivity and Optoelectronic Properties of Self-Powered Solar-Blind Ag_2O/β-Ga_2O_3 Heterojunction-Based Photodetector with Ag:AZO Co-Sputtered Electrode

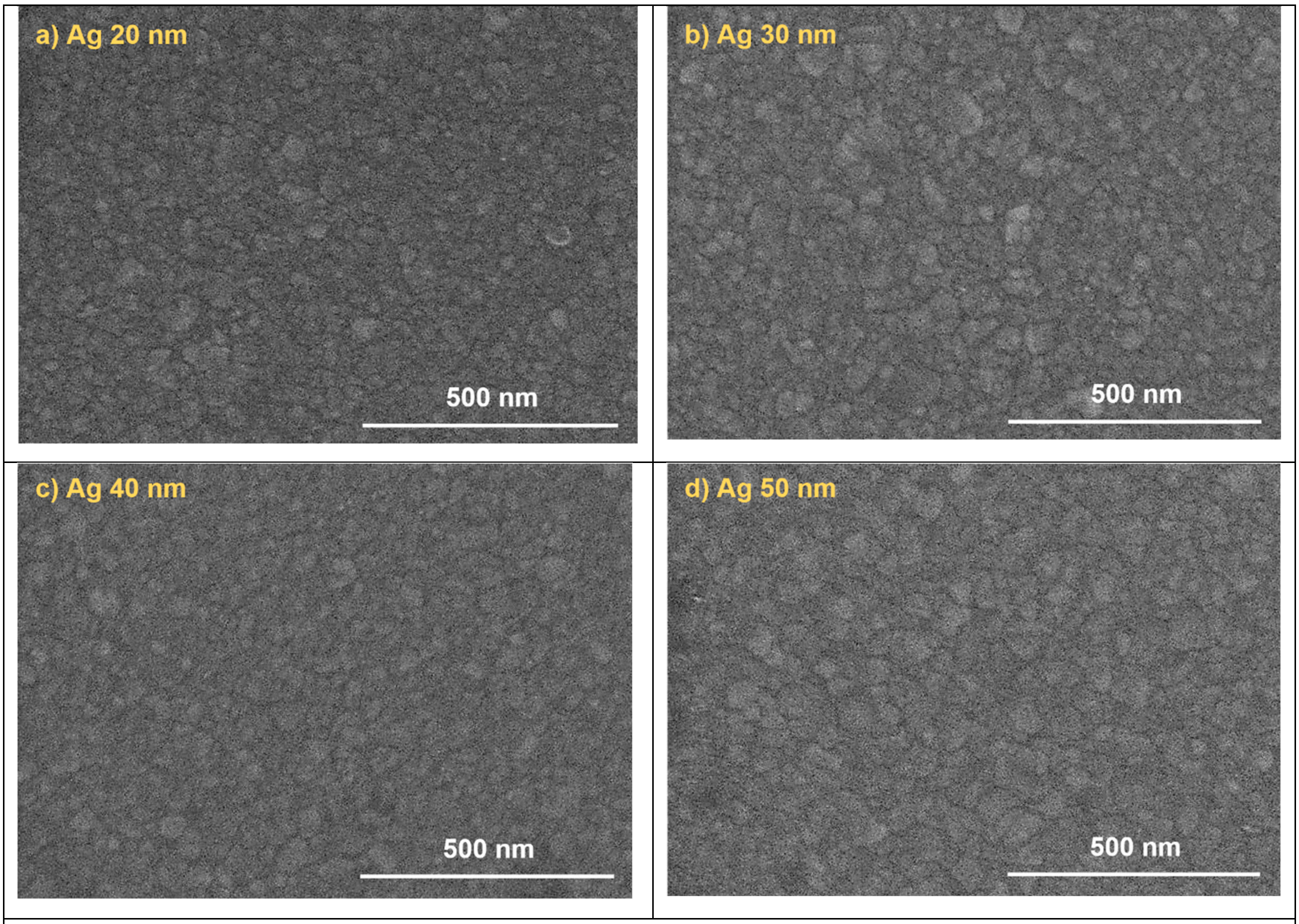

Figure S1 Thickness-dependent SEM images of Ag thin films

a) 20 nm b) 30 nm c) 40 nm d) 50 nm

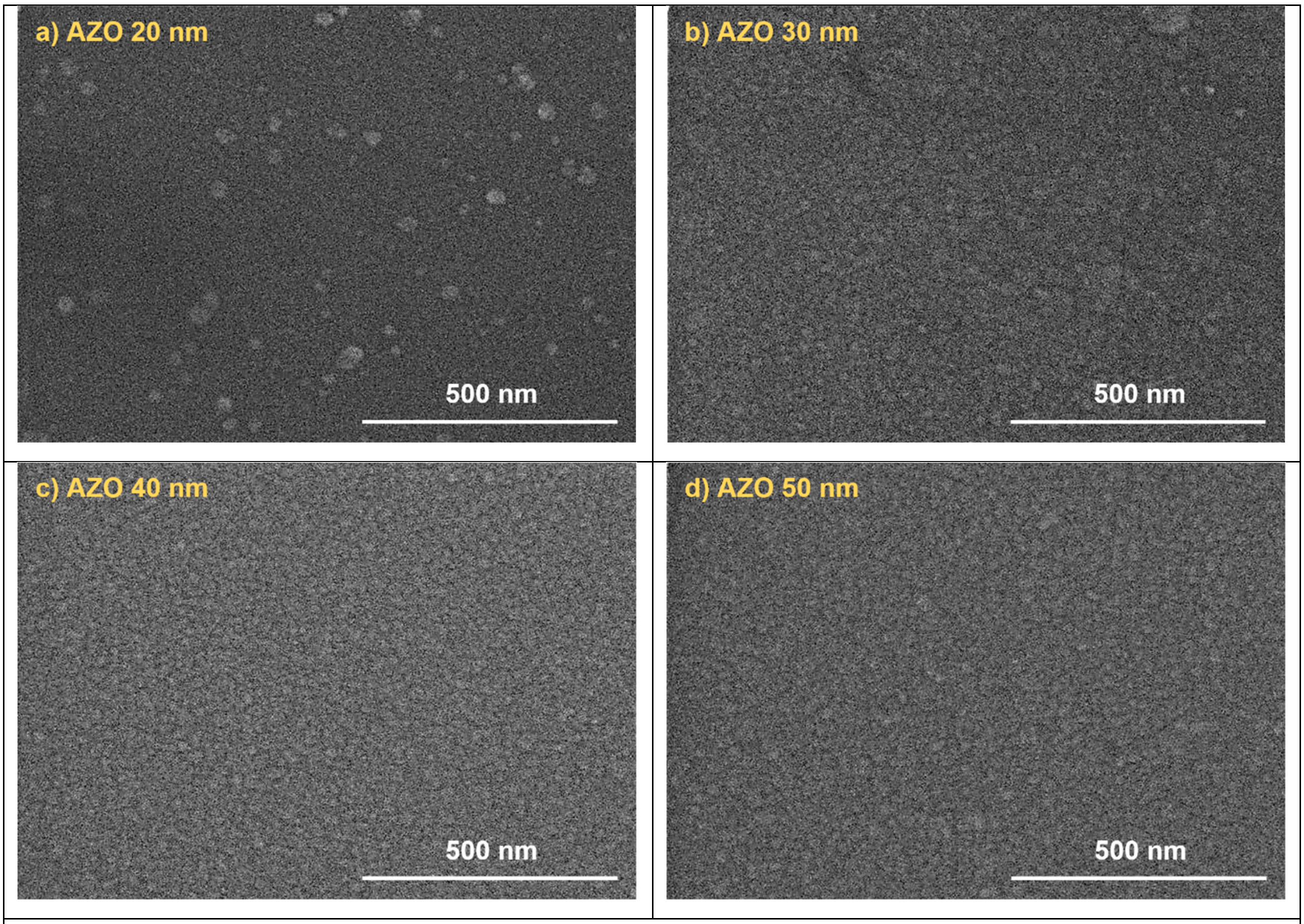

Figure S2 Thickness-dependent SEM images of AZO thin films

a) 20 nm b) 30 nm c) 40 nm d) 50 nm

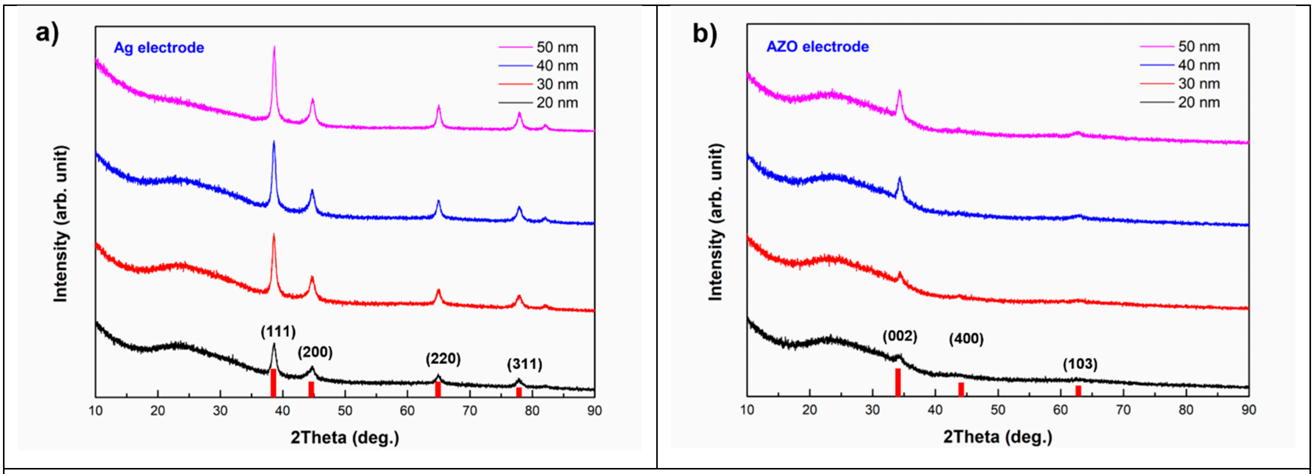

Figure S3 Structural characteristics of Ag:AZO thin film depending on thickness

a) XRD patterns of Ag b) XRD patterns of AZO

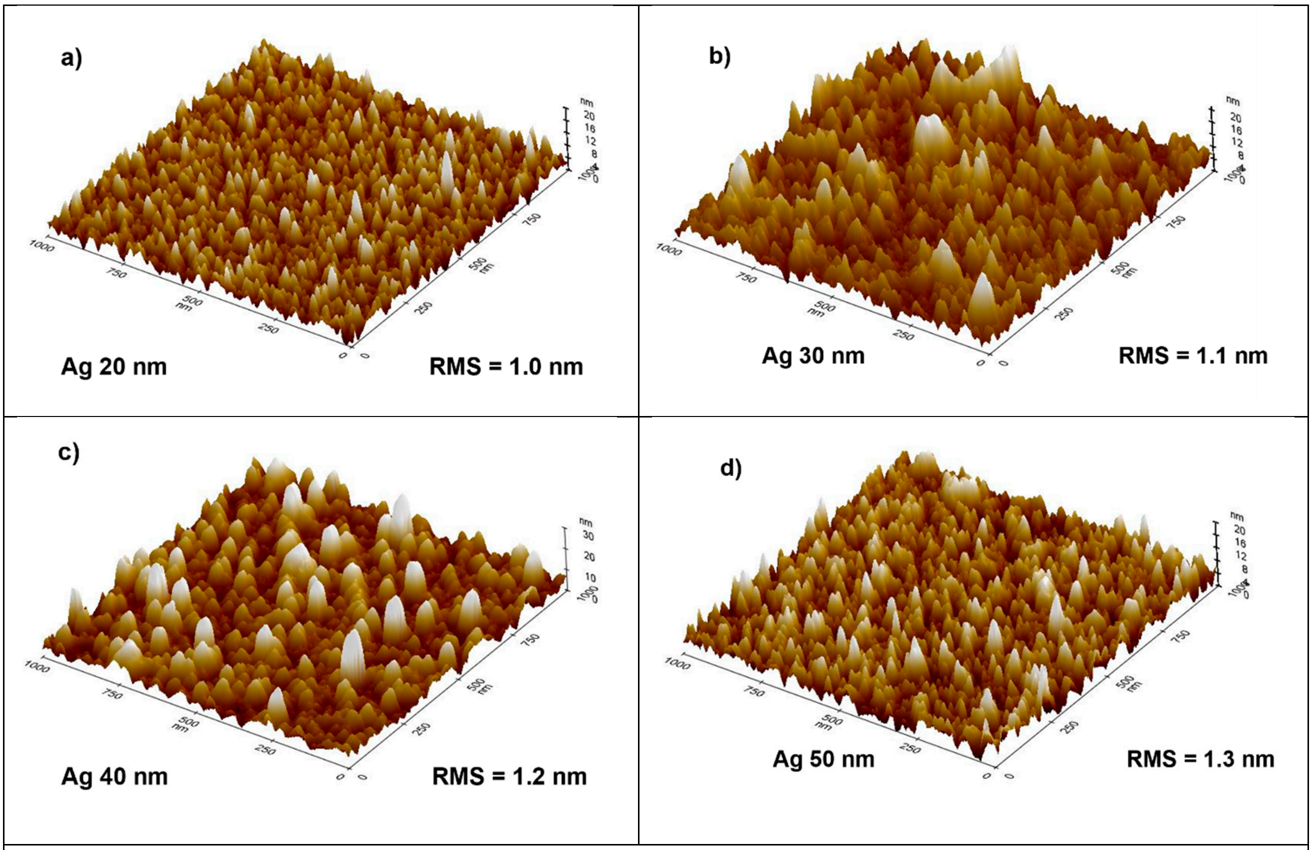

Figure S4 AFM images of the Ag thin film as a function of thickness
(a) 20 (b) 30 (c) 40 (d) 50 nm

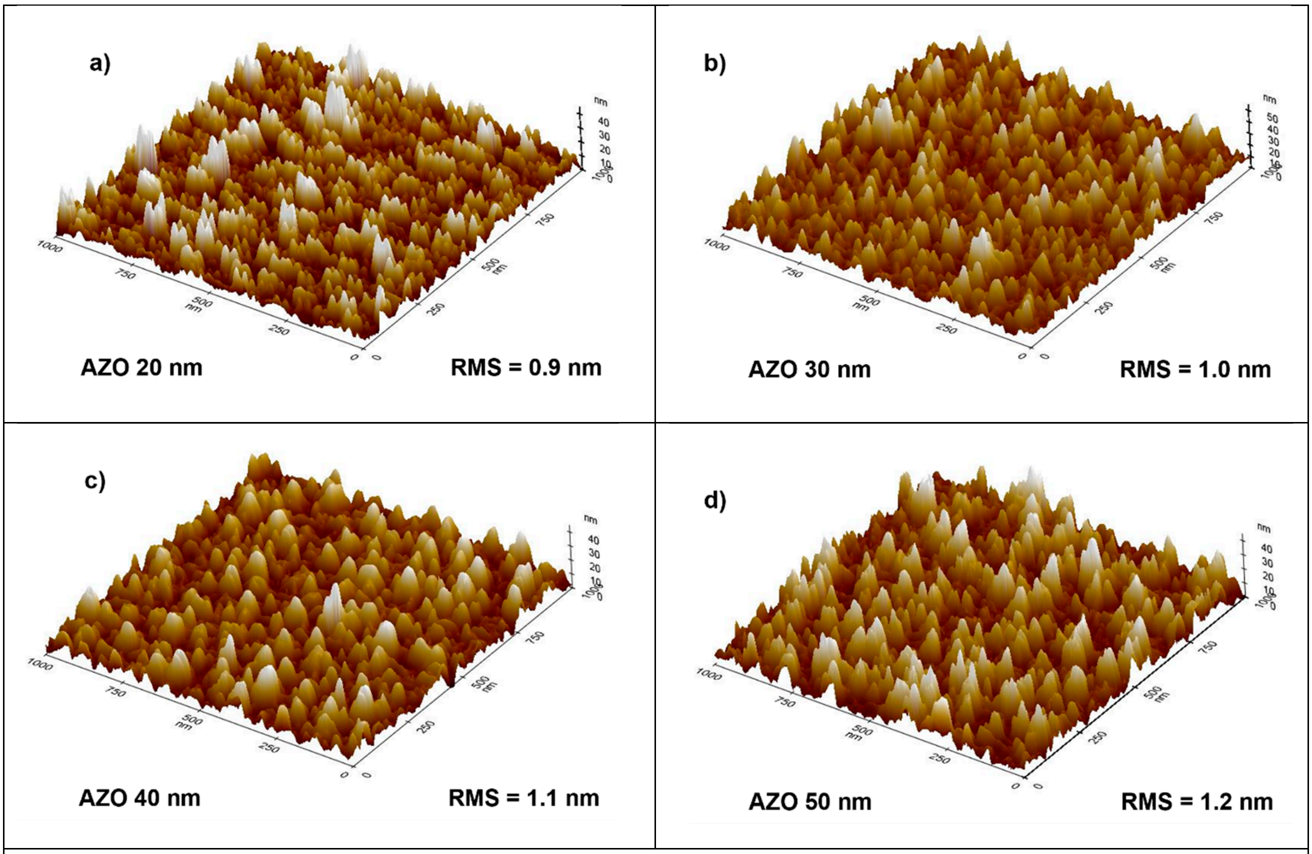

Figure S5 AFM images of the AZO thin film as a function of thickness
(a) 20 (b) 30 (c) 40 (d) 50 nm

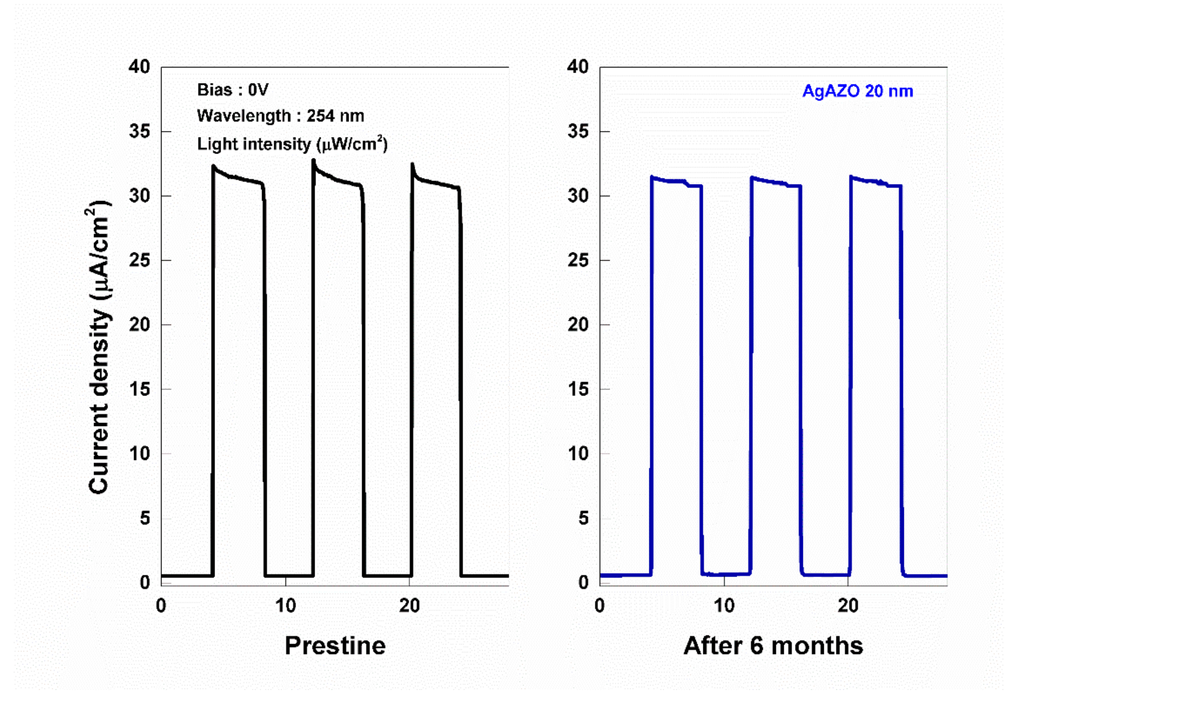

Figure S6. Sustainability of Ag:AZO photodetector after 6 months
